# Impacts of Genome-Wide Analyses on Our Understanding of Human Herpesvirus Diversity and Evolution

**DOI:** 10.1128/JVI.00908-17

**Published:** 2017-12-14

**Authors:** Daniel W. Renner, Moriah L. Szpara

**Affiliations:** a Department of Biochemistry and Molecular Biology, Center for Infectious Disease Dynamics, and the Huck Institutes of the Life Sciences, Pennsylvania State University, University Park, Pennsylvania, USA; University of California, Berkeley

**Keywords:** herpesvirus, bioinformatics, diversity, evolution, genetic recombination, genomics, minority variant, polymorphism, viral pathogenesis

## Abstract

Until fairly recently, genome-wide evolutionary dynamics and within-host diversity were more commonly examined in the context of small viruses than in the context of large double-stranded DNA viruses such as herpesviruses. The high mutation rates and more compact genomes of RNA viruses have inspired the investigation of population dynamics for these species, and recent data now suggest that herpesviruses might also be considered candidates for population modeling. High-throughput sequencing (HTS) and bioinformatics have expanded our understanding of herpesviruses through genome-wide comparisons of sequence diversity, recombination, allele frequency, and selective pressures. Here we discuss recent data on the mechanisms that generate herpesvirus genomic diversity and underlie the evolution of these virus families. We focus on human herpesviruses, with key insights drawn from veterinary herpesviruses and other large DNA virus families. We consider the impacts of cell culture on herpesvirus genomes and how to accurately describe the viral populations under study. The need for a strong foundation of high-quality genomes is also discussed, since it underlies all secondary genomic analyses such as RNA sequencing (RNA-Seq), chromatin immunoprecipitation, and ribosome profiling. Areas where we foresee future progress, such as the linking of viral genetic differences to phenotypic or clinical outcomes, are highlighted as well.

## INTRODUCTION

Herpesviruses infect and affect every human on the planet, with a universally penetrant global public health impact (1–4). Most adult humans carry one or more members of the nine herpesvirus families that infect our species. These include the alpha-subfamily members herpes simplex virus 1 and 2 (HSV-1/2 or human herpesvirus 1 and 2 [HHV-1/2]) and varicella-zoster virus (VZV or HHV-3); the beta-subfamily of human cytomegalovirus (HCMV or HHV-5) and human herpesviruses 6A, 6B, and 7 (HHV-6A/6B/7); and the gamma-subfamily of Epstein-Barr virus (EBV or HHV-4) and Kaposi's sarcoma-associated herpesvirus (KSHV or HHV-8) (5). Advances in our understanding of the molecular biology and evolution of these herpesviruses have been informed and advanced by work on other large DNA viruses such as poxviruses of humans and animals; multiple families of bacteriophage, baculoviruses, and other insect viruses; and amoebal giant viruses such as mimivirus (6).

**FIG 1:**
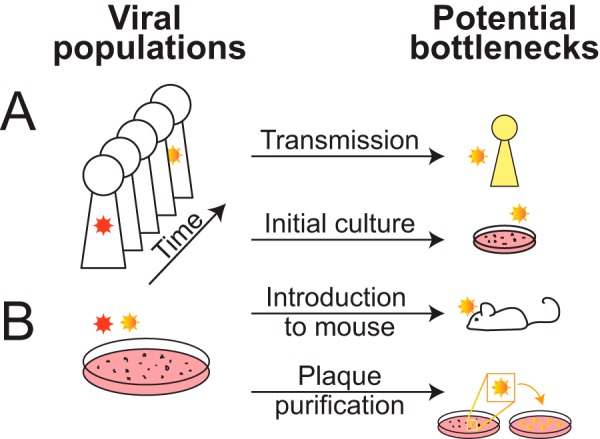
Opportunities for change in a given viral population arise both *in vivo* and *in vitro*. (A) The viral population in an infected individual (represented by red or red-shaded virion) may change over time due to immune selection or the accumulation of genetic drift. Bottlenecks at transmission to a new host or during introduction to tissue culture may allow a new genotype to become prevalent, akin to a genetic shift. (B) A viral stock grown in culture is also a viral population, which may undergo changes during introduction to an animal model or through plaque purification. See Fig. 2 for an expanded view of the genomes contained in the viral population.

**FIG 2:**
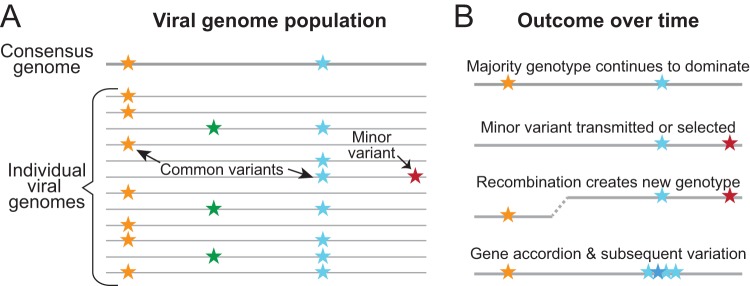
Viral genomes with subtle variations contribute to the overall viral population and enable change over time. A viral population may contain minor variants (A) that remain unnoticed until selective pressures or bottlenecks reveal them (B). Deep sequencing approaches can reveal minor variants in the overall viral population, but most HTS approaches report only the consensus genome population. The consensus genome is a summary of the most common variants (e.g., those indicated by orange and blue stars) found in a majority of the sequenced genomes, but that exact genotype does not necessarily predominate in nature. As shown in the exaggerated example in panel A, the consensus genome (thick gray line) contains variants that are found in the majority of genomes (thin gray lines) but that are found only rarely in the same genome. Minor variants or alleles (e.g., those indicated by green or orange stars) are not included in the consensus genome at all, but a transmission bottleneck or subsequent selective pressure may lead to a minor variant becoming the majority genotype in the future (B). Recombination can also create entirely new genotypes, which can become dominant through bottlenecks or external selective pressures. Gene accordions, as demonstrated in vaccinia virus, result from expansion and subsequent variation of a gene under strong selective pressure.

Here we review recent studies that have used high-throughput sequencing (HTS) and genome-wide analyses to explore the diversity and evolution of herpesviruses. In the first half of this review, we examine data suggesting that the diversity and evolution of herpesviruses are impacted by mechanisms extending beyond the usual consideration of polymerase fidelity. These include the influence of minority alleles and standing variation in the virus population, recombination between viral genomes, horizontal gene transfer, and nontemplated mechanisms such as ribosome frameshifting and RNA editing (Fig. 1 and 2). We also consider how these mechanisms of variation impact our ability to manipulate herpesviruses in culture. In the second half of this review, we summarize the tremendous gains in identifying the genetic diversity found among the members of each species of human herpesvirus. We explore how to accurately describe the viral populations that researchers handle experimentally. The necessity for a strong foundation of high-quality genomes—and the bioinformatics tools that enable their production—are also discussed. Many new insights have been enabled by the application of HTS and bioinformatics to herpesvirus genomes, and we end with the challenges that lie ahead.

## (IN)STABILITY OF LARGE DNA VIRUSES

The perception of most virologists is that RNA viruses are inherently variable and that DNA viruses are inherently stable (7, 8). At the molecular level, this view stems from the lack of error correction by most RNA-dependent RNA polymerases. In contrast to RNA viruses, most DNA viruses display high polymerase fidelity and error correction. For herpesviruses such as HSV-1, early studies of mutation rates focused on single genes and detected mutation rates in the range of ∼1 × 10^−7^ or ∼1 × 10^−8^ mutations per base per infectious cycle (9, 10). These rates are often quoted in comparisons of RNA viruses to DNA viruses (7, 8, 11) and are matched by restriction-fragment length polymorphism (RFLP) analyses comparing herpesviruses from disparate geographic locations (12). However, these data fail to explain the surprising ease with which herpesvirus variants can be selected or revealed (Fig. 1). For instance, drug-resistant mutations can be selected from drug-sensitive HSV-1 and HSV-2 populations at a rate of 1 in 10^4^ or 1 in 10^3^ PFU (13). This suggests that the rate of standing variation in the population in herpesviruses may be higher than previously appreciated (14) and/or that the different time scales of experimental settings versus evolutionary comparisons are at odds (15).

There are data to support both the hypothesis of standing variation and that of differing time scales. Using HTS approaches, several studies have described the existence and expansion of standing variation in HCMV populations in nonimmunocompetent hosts, such as congenitally infected infants and immunosuppressed or transplant patients (16–20). In a short-time-scale investigation of Muller's ratchet—the hypothesis that small asexual populations accumulate deleterious mutations—Jaramillo et al. took 10 individual subclones of HSV-1 and subjected each to repeated population bottlenecks through sequential plaque-to-plaque transfers (21). Two clonal lineages were completely lost during this process, and single-gene analysis of the remaining clones revealed a mutation frequency of 3.6 × 10^−4^ substitutions per base per plaque transfer. The authors also found reduced mortality after intracerebral inoculation into mice for these serially passaged clones (21). Even in the absence of intentional selective pressure, genome-wide HTS comparisons of HSV-1 and HCMV subclones have revealed nucleotide variations in up to 3% to 4% of the genome (22–25). A higher-than-expected frequency of observed mutations in herpesvirus populations was also found in a recent application of phylodynamic inference to DNA viruses, which estimated the substitution rate of HSV-1 to be ∼1 × 10^−5^ or ∼1 × 10^−4^ mutations per base per year (15). Many of the studies cited above have focused on HSV-1 as a model herpesvirus, but there may well be subfamily- or species-specific differences in the amount of standing variation in the population, the number of replicative cycles per year, and/or the selection pressures faced during viral transmission in real-world settings. Innovative combinations of genome-wide HTS applications, with models that account for positive selection and standing variation, will be needed to bring these diverse data into synchrony.

## RECOMBINATION AS A DRIVING FORCE IN DNA VIRUS EVOLUTION

Mutation and evolution in herpesviruses result not only from base substitutions but also from recombination between strains and, to a lesser extent, between species. Recombination in herpesviruses can provide a driving force for evolutionary shifts, akin to that associated with reassortment in segmented RNA viruses (26). HTS studies of laboratory-generated recombinants of HSV-1 have revealed a bias toward breakpoints being detected in repetitive tracts, intergenic regions, and areas of higher G+C content (27). However, most studies of recombination in human herpesviruses have focused on naturally circulating variants and have inferred historical sites of recombination and phylogenetic relationships from the comparison of disparate strains. For example, increased genomic surveillance of VZV has expanded the number of known phylogenetic clades for this species in recent years; this has provided evidence for ancient, interclade recombination as well as for modern recombination between individual strains (28–32). For the beta-herpesvirus HCMV, multiple groups have confirmed the finding of rampant genome-wide recombination among different HCMV strains (19, 33, 34). Lassalle et al. focused most deeply on this aspect of HCMV evolution and found that particular sections or islands of the HCMV genome appeared to cosegregate, whereas widespread recombination between strains was detected everywhere else in the genome (34). The authors postulated that genes in these islands are codependent, thus enabling higher fitness and a selective advantage for genomes that lack recombination events inside these regions.

In the case of the alpha-herpesvirus HSV-1, there is evidence of rampant recombination between different isolates or strains, but not yet sufficient data to discern whether the recombination events are ancient or extant (35, 36). Evidence for ancient recombination between HSV-1 and the distantly related species HSV-2 has been found by two separate groups (37, 38), who recently described several loci in the HSV-2 genome that contained HSV-1-like DNA. This inference is based on the high similarity of these regions to extant HSV-1 genomes and on their divergence from a unique and apparently historical HSV-2 genotype that has thus far been found only in Africa (39, 40). Additional evidence of modern recombination in natural settings stems from the veterinary herpesvirus literature (41). In 2012, Lee and colleagues demonstrated that a virulent avian herpesvirus that had created an outbreak in Australian poultry was in fact a spontaneous recombinant resulting from two live-attenuated vaccines that were both in use (42). This example is bolstered by multiple others among the veterinary herpesviruses which have been reviewed recently elsewhere (41). The next challenge for understanding herpesvirus recombination events is to address where and how often they occur *in vivo*, since recombination requires the co-occurrence of two distinct viral genomes in a single cell of the same host—an event which may be rare and difficult to detect in clinical or field settings.

## LOSING OR GAINING GENE FUNCTIONS IN CULTURE

Our ability to conduct experimental studies of herpesviruses, and to develop therapeutics and vaccines, depends vitally on cell culture techniques. However, cell culture can induce unintentional selective pressure on viral populations, as has been recognized most notably in the case of HCMV. Prior studies revealed that laboratory strains of HCMV such as AD169 and Towne had not only accumulated minor changes associated with genetic drift but also lost multiple genes during their adaptation to cell culture (22, 24, 43). The regions lost *in vitro* had functions associated with cell tropism and immune evasion *in vivo*. Although no similar link between frequent deletions and loss of *in vivo*-specific functions has yet been discovered for other herpesviruses, a tendency for loss of specific genomic regions has been observed. Frequent deletion of the UL55-UL56 region has been observed in cultured HSV-1 strains, although the phenotypic impact of this loss is unknown (36). The loss of genomic regions has also been demonstrated in other large DNA viruses such as mimivirus, which undergoes gene loss from its termini during repeated passage in amoebal culture (44). There is a pressing need to document the nature of any changes that occur during herpesvirus introduction to culture and subsequent passages thereafter, so that the accumulation of genetic drift and/or the selective pressures of cell culture can be better understood.

Although viral propagation in cell culture can induce the loss of gene functions required for *in vivo* growth, it can also facilitate experimental insights by revealing transient genome intermediates in the process of viral adaptation. This was demonstrated in a recent study using HTS and comparative genomics in the poxvirus vaccinia virus (VACV), which relies on two viral antagonists to combat the host antiviral protein kinase R (PKR) (45). After experimental deletion of one viral PKR inhibitor, the viral genome population developed an accordion-like expansion of the other inhibitor (Fig. 2B). Variations then arose and were positively selected in the extra copies of this PKR antagonist. These variants tended to remain in the progeny viral population even when the accordion-like gene array collapsed. The examination of genome content after each round of viral replication in culture revealed the existence of these intermediates in viral evolution. These data raise the intriguing question of whether similar mechanisms could occur in herpesviruses. Further investigation of herpesvirus adaptation to selective pressure, with analyses performed at frequent intervals throughout positive selection, will be required to test whether intermediates of viral evolution can be detected for herpesviruses as well.

## HOST-VIRUS HORIZONTAL GENE TRANSFER

Horizontal gene transfer (HGT), or the movement of genetic material between unrelated organisms, provides another avenue for evolutionary adaptation. In the case of herpesviruses, at least 20% of the core genes shared by all herpesvirus subfamilies are surmised to have cellular origins, while others appear to have originated in another viral species (46–50). The specific source, mechanism, and timing of these ancient HGT events in herpesvirus evolutionary history are not known. Most herpesviruses do not integrate into the host genome during replication. The gamma-herpesvirus EBV can be found occasionally in an integrated state, although it is not a required aspect of its life cycle (51, 52). However, human HHV-6A and HHV-6B and Marek's disease virus, an alpha-herpesvirus of poultry, do integrate into host telomeres as a regular part of their life cycle (53–56). The germ line or chromosomal integration of human herpesviruses (ciHHV), usually HHV6A, is detected in about 1% of the human population (51, 53–56). Recent data from baculovirus-moth model systems indicate that HGT between host and virus does not require viral integration and excision from the host genome (57, 58). Instead, Gilbert et al. found that HGT can be mediated by transposable elements (TEs) that move between host and viral genomes (57) and that recombination of host DNA into viral progeny can occur at sites of microhomology between the host and viral genomes (58). Baculovirus genomes with integrated host DNA constituted only about 5% of the viral progeny and did not remain in the population beyond a few cycles of replication, suggesting that these are transient intermediates with deleterious fitness effects (58). Nonetheless, these data illustrate a potential avenue for evolutionary HGT in nonintegrating viruses and suggest that it may be of interest to screen for signs of host DNA integration into progeny herpesvirus genomes.

## OTHER CONTRIBUTIONS TO FUNCTIONAL DIVERSITY

Mechanisms of genetic variation such as single-nucleotide changes, recombination, and horizontal gene transfer are well accepted for their roles in the evolution of herpesviruses. Limited but exciting data suggest that other mechanisms, including several that are more often associated with RNA viruses, may also contribute to the diversity of herpesvirus coding potential. These include ribosome slippage, RNA editing, and novel transcripts revealed by RNA sequencing (RNA-Seq) and ribosome profiling or footprinting. These mechanisms may not be revealed by examining populations of viral genomes but may nonetheless influence phenotypes observed *in vivo*.

Ribosome frameshifting and RNA editing are two mechanisms by which herpesviruses can achieve a phenotypic outcome different from the outcome that would be predicted by analysis of the nucleotides encoded in their genome. These outcomes can be detected by examining viral transcripts or proteins but may otherwise go undetected in the comparison of genome sequences. Ribosome frameshifting is a regular feature of translation for retroviruses such as HIV, where it enables the production of nucleocapsid and polymerase from the same RNA transcript. Although it is less frequent, ribosome frameshifting has been demonstrated to occur on transcripts of thymidine kinase (TK) in HSV-1 (59, 60). Microdeletions at homopolymers in the TK or polymerase genes of HSV-1 are a common route of viral escape from the activity of the antiviral drug acyclovir (61, 62), and ribosome frameshifting of defective transcripts in these drug-resistant genomes allows production of a low level of functional protein (59, 60). RNA editing or transcriptional stuttering is another mechanism better associated with RNA viruses which is used to generate more than one transcript from a single open reading frame. RNA editing has recently been demonstrated to occur in the gamma-herpesviruses EBV and KSHV, where it affects microRNAs (EBV) or viral protein-coding genes (KSHV) (63, 64). The phenotypic impacts of these RNA editing events remain to be determined.

Finally, HTS approaches have highlighted the presence of previously unrecognized coding potential in herpesvirus genomes, through the use of RNA-Seq and ribosome profiling approaches that demonstrate the shift from host to viral transcriptional and translational control during infection (64–68). These approaches have illuminated new transcripts and coding potential in HCMV, EBV, and KSHV (64–67) and demonstrated the disruption of transcript termination in HSV-1 (68). The novel transcripts found in these studies are too new to have been considered in prior comparative genomics analyses, but future studies may reveal their influence on the biology and evolution of these herpesviruses.

## CAPTURING AND CATALOGING THE DIVERSITY OF HERPESVIRUSES

Early applications of HTS to herpesvirus genomes focused on just one or two examples of a given species (69), using viral strains that had been previously characterized in cell culture. The norm for HTS studies has now shifted to include either comparisons of a large number of viral strains at a time or a deeper investigation of viral setting or outbreak. This expansion of known diversity has driven the definition of new phylogenetic clades and facilitated the reconstruction of the evolutionary history of VZV (28, 30–32), HSV-1 (23, 36, 70–72), HSV-2 (73–76), HCMV (20, 33, 34, 77), HHV 6A/6B (119), EBV (78, 79), and KSHV (80), as well as animal herpesviruses (41). While it is clear that increasing the number of fully sequenced genomes for each species widens our knowledge of viral diversity, the next challenge lies in dissecting the phenotypic impacts of the observed genetic differences in these viral populations. Achieving that goal will require the integration of phenotypic measures of viral fitness, with fully sequenced viruses and comparative genomics, to infer how specific genetic differences influence the outcome of infection.

As the genomic comparison of large DNA viruses from cultured stocks became more tractable, the goal of achieving similar resolution from uncultured viruses became a priority. The development of oligonucleotide enrichment methods has facilitated this goal for herpesviruses (29, 37, 77, 81). Oligonucleotide enrichment uses the known genomes of cultured viruses to design small RNA- or DNA-based probes or baits that can hybridize with sparse amounts of the targeted viral genomes in any mixed sample. These hybridized fragments are then isolated using a tag such as biotin on the synthetic oligonucleotide baits. Once enriched from a mixed source sample, the viral genome fragments can be amplified and sequenced using standard HTS approaches. Oligonucleotide enrichment has enabled the capture of herpesvirus genomes from saliva, blood, skin swabs, vesicle fluid, and more (29, 37, 81). Improvements in the isolation and handling of ancient DNA, combined with oligonucleotide enrichment, have even demonstrated the feasibility of recovering historical samples, such as the recent genome sequencing of 17th century smallpox (variola) DNA from mummified human remains (82). This type of ancient viral genome recovery has not yet been attempted for a herpesvirus, but if the challenge is surmounted it may similarly illuminate the rate of evolution and local adaptation seen in these viruses.

## THE CONSENSUS VERSUS THE MINORITY

Many of the studies described above that catalogued herpesvirus diversity have focused on defining the consensus genome of each new sample. The consensus genome represents the most common allele or nucleotide at each position (Fig. 2). In the simplest case, the consensus genome is derived from the most common member of the viral population. However, the consensus genome may not exist in nature as the most common genome format—in other words, it may be an amalgamation of several genotypes that exist separately but not together on a single genome (Fig. 2A). For small viruses, the ability to clone and determine the genotypes of individual genomes has enabled the modeling of viral populations and the development of software that can infer likely haplotypes from HTS data (see reference 83 for a review). Barcoded HTS methods offer the potential to improve haplotype linking for large DNA viruses, but these have not yet been widely applied to human herpesviruses. Thus far, no HTS method has proven capable of sequencing individual strands of DNA viruses that are >100 kb in length with sufficient accuracy and reproducibility to make enable the comparison of individual genomes in a viral population. Limited applications of nanopore-based sequencing (MinION; Oxford Nanopore) or single-molecule real-time sequencing (SMRT; Pacific Biosystems) to herpesviruses have demonstrated the potential of these methods (71, 84, 85). However, both technologies currently suffer from error-prone sequence reads, leading to limited applications during this phase of technical development. For these reasons, most researchers using Illumina or other short-read HTS platforms have focused on detecting the location and prevalence of minority variants, without attempting to link their co-occurrence on individual genomes (i.e., determining haplotypes).

As for RNA virus genomes, deep coverage of large DNA virus genomes has enabled the detection of minority variants, or heterogeneous alleles, and their potential expansion in different environments (Fig. 2). It was recently demonstrated that bottlenecks and selective sweeps occur during human congenital infection by the beta-herpesvirus HCMV. In a series of papers, Renzette et al. showed that HCMV genomes sequenced from the urine of congenitally infected infants harbored multiple loci with minority variants (17–19). While the authors initially posited that HCMV diversity approached the level of a quasispecies, their subsequent modeling suggested that viral diversity in these congenitally infected infants resulted from a combination of sources such as reinfection, recombination, positive selection, and bottlenecks during intrahost transmission between body sites (19, 86, 87) (Fig. 2B). Other groups have found a lower level of intrahost diversity in the context of noncongenital HCMV infections (16, 20, 33, 34, 77), suggesting that congenital infections may represent a special case for viral diversification. Recently, HTS was applied to detect low-frequency drug resistance mutations in the HCMV genome, which has confirmed the potential impacts of intrahost viral diversity on clinically important outcomes such as drug resistance (20). Although the study was conducted retrospectively, after patient treatment, the potential application for real-time HTS screening of viral populations during patient treatment is clear (88, 89).

## VIRAL ISOLATES, STRAINS, VARIANTS, AND SUBCLONES

HTS methods have brought the issue of viral population diversity to the fore. Almost all experiments conducted with herpesviruses utilize a population of genome-containing virions (Fig. 2). The same is true of most samples collected from a host source. Because viral populations can shift over time or through handling (Fig. 1), it is crucial to clearly define each viral population under study and to know its history (24). A virus collected from a point source at a specific time is often called an isolate, and it may be referred to as a strain after its growth and expansion in culture. Even after being established as a strain in cell culture, a viral population may still undergo further change (Fig. 1). This can occur through random genetic drift, during intentional bottlenecks such as plaque purification, or through the generation of transgenic or mutant subclones. A viral strain can thus consist of a mixed population of viruses, or it may have undergone a bottleneck that led to the creation of a homogeneous population. For instance, the HSV-1 strain KOS has developed variants by genetic drift over passages in culture, as well as through intentional plaque purification of subclones (25, 90–94). These variants and subclones differ in observable phenotypes such as their ability to elicit Toll-like-receptor (TLR)-dependent immune responses (93), pathogenesis in animal models of HSV encephalitis (91), and expression of antigenic proteins (90, 92). This example emphasizes the importance of knowing the identity and history of the viral populations used in all laboratory experiments.

A more consistent standard of description for viral populations would benefit the herpesvirus community. Descriptive terms such as “clinical isolate” and “laboratory strain” are often used to refer to the low (clinical) versus high (laboratory strain) number of passages that a viral population has undergone in cell culture—though in practice these terms are interpreted differently by every research group (5, 24). There is no historical standard for whether or not a herpesvirus isolate should be plaque purified before it can be called a strain. There is no common term used to describe viral genomes that are collected and sequenced directly from a host, without amplification in culture—these are often referred to simply as genomes, sequences, or genotypes (29, 75, 77, 95). The VZV research community and others have moved toward a viral naming system, akin to those utilized for RNA viruses and bacteria, which includes both virus species and host species and preserves data on sample origin (e.g., geographic location), year of isolation, and the name(s) of the strain, variant, or subclone (96, 97). Following on the HSV-1 KOS example above, one variant of the strain is named HSV-1 Homo sapiens/Texas, USA/1963/KOS-KOS63, indicating both the year and location of its origin (25, 94). This approach may help to alleviate current issues in comparing data across laboratories, where viruses of the same common name (e.g., HSV-1 KOS or HCMV AD169) may differ in both genotype and phenotype (24, 25, 94).

## BIOINFORMATICS AND THE FOUNDATION OF HIGH-QUALITY GENOMES

The ability to rapidly sequence and assemble large DNA virus genomes has been facilitated by advances in software and computational workflows, although the diversity of options and different standards of publication have led to a wide variety of finished-genome qualities. One major choice underlying all HTS genome analysis is whether to build new viral genomes by alignment of reads to a prior reference genome or by *de novo* assembly. Alignment-based approaches utilize prior genome knowledge to achieve a faster outcome, but these are prone to miscalling of minority and structural variants (83). *De novo* assembly is unbiased by prior data and can more easily detect new variants and structural differences, but it is more computationally intensive and can entail the need for more input to curate the genomes thereafter (83). Open-source options for viral genome analysis and annotation include Web-based platforms and those using command-line (Unix-like) interfaces. The vast majority of viral *de novo* assembly algorithms have been developed and tested only for RNA viruses (see reference 83 for a review of options). We developed the Web-based viral *de novo* assembly workflow VirAmp specifically for herpesviruses (98), using the Galaxy framework of Web-accessible bioinformatics tools (99, 100). Most other options for viral genome assembly, alignment, annotation, and comparison rely on a Unix command-line interface, which requires more skill to operate. Unix-based software options are freely available through repositories such as BitBucket and GitHub and include programs such as the *de novo* viral genome assembly workflow VirGA (23), the aligners Bowtie and BWA (83), and the structural variant detector Wham (101). Researchers can also choose from commercial packages that offer one-button solutions for alignment or *de novo* assembly, such as Geneious (Biomatters) and CLC Bio (Qiagen) (99, 100). These options have made complex bioinformatics tasks accessible to a wider audience, to the extent that whole-genome sequencing and comparisons of diverse bacteriophage are now part of the undergraduate science education curriculum at many universities (102, 103).

The rationale for a strong foundation of high-quality genomes has been well established by the human genome project and multiple microbial genome projects (104). A wide range of secondary HTS applications, such as RNA-Seq, ribosome profiling, chromatin-immunoprecipitation (ChIP) sequencing, and chromatin conformation capture (CCC or 3C) assays, rely on the accuracy of initial genome sequences (105). Use of a misassembled or poorly annotated viral genome leads to errors in these secondary analyses. Similarly, mapping data from downstream analyses of one viral strain onto the reference genome of another strain can produce misleading outcomes. Gaps or unfinished regions in genome assemblies also create an issue, since these create missing data in all subsequent comparative genomics approaches. Publications occasionally omit the deposition of intact genome sequences, limiting future comparisons of these data (35, 71, 106). The failure to complete the sequence of genes with complex tandem repeats or G/C-rich sequences means that these genes are often excluded from comparative genomics studies or are represented by a far smaller number of examples (see, for example, references 36, 73, 75, and 107). Incomplete intergenic regions can skew the assessment of overall genomic diversity, since genetic drift tends to accumulate in intergenic regions. Unresolved gaps also prohibit any insight from secondary analyses such as RNA-Seq or ChIP in these regions, since data cannot be mapped to these areas. The tremendous insights to be gained from HTS technologies and all of their secondary applications thus rely on a strong foundation in the initial deciphering of viral genome populations.

## FUTURE DIRECTIONS

Here we have focused on the several areas of recent progress in understanding the genomic diversity and evolution of human herpesviruses. These advances have been driven by the rapid expansion and application of HTS, bioinformatics, and comparative genomics in virology. Together, these data have reshaped our sense of the stability of herpesvirus genomes. While these viruses possess high-fidelity polymerases, their ability to accrue standing variation, and to undergo recombination with neighboring genomes, creates many opportunities for selective pressures to induce rapid genetic shifts. Examples of this include the expansion of minority variants in niche locations in congenitally infected infants and the selection of drug-resistant variants during antiviral therapy (16, 19, 20, 86). In addition to these recent advances and insights, we foresee several areas of future promise.

First, we foresee the improvement and extension of third-generation sequencing and genome editing technologies to herpesviruses. Early applications of MinION and SMRT long-read sequencing to herpesvirology have shown promise in revealing novel transcriptional networks (67, 85) and in confirming a new synthetic genome approach to introduce multiple simultaneous changes to a herpesvirus genome (84). These third-generation sequencing methods may also enable the detection of methylated bases, secondary structures, and even substrates besides DNA (105). As the accuracy of these methods improves, we foresee their use to advance the detection of recombinant genomes and structural variants, as well as to define haplotypes in mixed populations (Fig. 2). The advances in clustered regularly interspaced short palindromic repeat (CRISPR)-Cas systems for genetic engineering of herpesviruses also represent an exciting area for future expansion (108–110). CRISPR-Cas approaches promise to speed the construction of viral mutants for reverse genetic studies (108, 109) and may have therapeutic potential for herpesvirus genome clearance (110). We also anticipate that HTS and genomic analyses of CRISPR-engineered viruses will be a fruitful way to confirm the desired genomic edits and rule out any off-target or bystander changes.

Finally, we consider the linking of viral genetic variation to observable phenotypes to be one of the greatest challenges for virology. The advance of HTS and genomics has begun to enable the application of genome-wide association studies (GWAS) and quantitative trait locus (QTL) approaches to herpesviruses (111, 112). Brandt and colleagues recently demonstrated the application of viral QTL mapping to HSV-1, by examining how specific viral genotypes contributed to phenotypes of ocular infection in mice (113, 114). That QTL study used the recombinant viral progeny of two attenuated strains of HSV-1, with the differing genetic composition of each recombinant being mapped to the nucleotide resolution level using HTS and comparative genomics (27). These forward genetic approaches complement prior decades of reverse genetic approaches, which established the function of herpesvirus genes and began to dissect the impacts of individual genetic variants (115, 116). However, the occurrence of gene deletions and genetic variations in living humans can be quite distinct from those seen in laboratory-constructed mutants (33, 75, 78), and there is significant interest in determining if and how these viral genetic variants may impact human clinical outcomes (79, 117, 118). This motivates the future extension of GWAS analyses to naturally circulating viral variants and clinical isolates. This will shed light on how viral genetic diversity intersects with human genetic differences to produce the spectrum of observed disease.

## References

[1] Looker KJ, Magaret AS, May MT, Turner KME, Vickerman P, Gottlieb SL, Newman LM 2015 Global and regional estimates of prevalent and incident herpes simplex virus type 1 infections in 2012. PLoS One 10:e0140765. doi:10.1371/journal.pone.0140765. 26510007 PMC4624804

[2] Looker KJ, Magaret AS, Turner KME, Vickerman P, Gottlieb SL, Newman LM 2015 Global estimates of prevalent and incident herpes simplex virus type 2 infections in 2012. PLoS One 10:e114989. doi:10.1371/journal.pone.0114989. 25608026 PMC4301914

[3] Jansen MAE, van den Heuvel D, Bouthoorn SH, Jaddoe VWV, Hooijkaas H, Raat H, Fraaij PLA, van Zelm MC, Moll HA 2016 Determinants of ethnic differences in cytomegalovirus, Epstein-Barr virus, and herpes simplex virus type 1 seroprevalence in childhood. J Pediatr 170:126–134.e6. doi:10.1016/j.jpeds.2015.11.014. 26707579

[4] Gantt S, Orem J, Krantz EM, Morrow RA, Selke S, Huang M-L, Schiffer JT, Jerome KR, Nakaganda A, Wald A, Casper C, Corey L 24 2 2016 Prospective characterization of the risk factors for transmission and symptoms of primary human herpesvirus infections among Ugandan infants. J Infect Dis doi:10.1093/infdis/jiw076. PMC4907408 26917575

[5] Davison AJ 2010 Herpesvirus systematics. Vet Microbiol 143:52–69. doi:10.1016/j.vetmic.2010.02.014. 20346601 PMC2995426

[6] Koonin EV, Krupovic M, Yutin N 2015 Evolution of double-stranded DNA viruses of eukaryotes: from bacteriophages to transposons to giant viruses: Evolution of dsDNA viruses of eukaryotes. Ann N Y Acad Sci 1341:10–24. doi:10.1111/nyas.12728. 25727355 PMC4405056

[7] Sanjuán R, Nebot MR, Chirico N, Mansky LM, Belshaw R 2010 Viral mutation rates. J Virol 84:9733–9748. doi:10.1128/JVI.00694-10. 20660197 PMC2937809

[8] Sanjuán R, Domingo-Calap P 2016 Mechanisms of viral mutation. Cell Mol Life Sci 73:4433–4448. doi:10.1007/s00018-016-2299-6. 27392606 PMC5075021

[9] Hall JD, Almy RE 1982 Evidence for control of herpes simplex virus mutagenesis by the viral DNA polymerase. Virology 116:535–543. doi:10.1016/0042-6822(82)90146-5. 6278726

[10] Drake JW, Hwang CBC 2005 On the mutation rate of herpes simplex virus type 1. Genetics 170:969–970. doi:10.1534/genetics.104.040410. 15802515 PMC1450398

[11] Drake JW 1991 A constant rate of spontaneous mutation in DNA-based microbes. Proc Natl Acad Sci U S A 88:7160–7164. doi:10.1073/pnas.88.16.7160. 1831267 PMC52253

[12] Sakaoka H, Kurita K, Iida Y, Takada S, Umene K, Kim YT, Ren CS, Nahmias AJ 1994 Quantitative analysis of genomic polymorphism of herpes simplex virus type 1 strains from six countries: studies of molecular evolution and molecular epidemiology of the virus. J Gen Virol 75:513–527. doi:10.1099/0022-1317-75-3-513. 8126449

[13] Sarisky RT, Nguyen TT, Duffy KE, Wittrock RJ, Leary JJ 2000 Difference in incidence of spontaneous mutations between herpes simplex virus types 1 and 2. Antimicrob Agents Chemother 44:1524–1529. doi:10.1128/AAC.44.6.1524-1529.2000. 10817703 PMC89907

[14] Renzette N, Gibson L, Jensen JD, Kowalik TF 2014 Human cytomegalovirus intrahost evolution—a new avenue for understanding and controlling herpesvirus infections. Curr Opin Virol 8:109–115. doi:10.1016/j.coviro.2014.08.001. 25154343 PMC4195796

[15] Firth C, Kitchen A, Shapiro B, Suchard MA, Holmes EC, Rambaut A 2010 Using time-structured data to estimate evolutionary rates of double-stranded DNA viruses. Mol Biol Evol 27:2038–2051. doi:10.1093/molbev/msq088. 20363828 PMC3107591

[16] Görzer I, Guelly C, Trajanoski S, Puchhammer-Stöckl E 2010 Deep sequencing reveals highly complex dynamics of human cytomegalovirus genotypes in transplant patients over time. J Virol 84:7195–7203. doi:10.1128/JVI.00475-10. 20463084 PMC2898262

[17] Renzette N, Bhattacharjee B, Jensen JD, Gibson L, Kowalik TF 19 5 2011 Extensive genome-wide variability of human cytomegalovirus in congenitally infected infants. PLoS Pathog doi:10.1371/journal.ppat.1001344. PMC3098220 21625576

[18] Renzette N, Gibson L, Bhattacharjee B, Fisher D, Schleiss MR, Jensen JD, Kowalik TF 26 9 2013 Rapid intrahost evolution of human cytomegalovirus is shaped by demography and positive selection. PLoS Genet doi:10.1371/journal.pgen.1003735. PMC3784496 24086142

[19] Renzette N, Pokalyuk C, Gibson L, Bhattacharjee B, Schleiss MR, Hamprecht K, Yamamoto AY, Mussi-Pinhata MM, Britt WJ, Jensen JD, Kowalik TF 6 7 2015 Limits and patterns of cytomegalovirus genomic diversity in humans. Proc Natl Acad Sci U S A doi:10.1073/pnas.1501880112. PMC4522815 26150505

[20] Houldcroft CJ, Bryant JM, Depledge DP, Margetts BK, Simmonds J, Nicolaou S, Tutill HJ, Williams R, Worth AJJ, Marks SD, Veys P, Whittaker E, Breuer J 9 9 2016 Detection of low frequency multi-drug resistance and novel putative maribavir resistance in immunocompromised pediatric patients with cytomegalovirus. Front Microbiol doi:10.3389/fmicb.2016.01317. PMC5016526 27667983

[21] Jaramillo N, Domingo E, Munoz-Egea MC, Tabares E, Gadea I 2013 Evidence of Muller's ratchet in herpes simplex virus type 1. J Gen Virol 94:366–375. doi:10.1099/vir.0.044685-0. 23100362

[22] Bradley AJ, Lurain NS, Ghazal P, Trivedi U, Cunningham C, Baluchova K, Gatherer D, Wilkinson GWG, Dargan DJ, Davison AJ 2009 High-throughput sequence analysis of variants of human cytomegalovirus strains Towne and AD169. J Gen Virol 90:2375–2380. doi:10.1099/vir.0.013250-0. 19553388 PMC2885757

[23] Parsons LR, Tafuri YR, Shreve JT, Bowen CD, Shipley MM, Enquist LW, Szpara ML 2015 Rapid genome assembly and comparison decode intrastrain variation in human alphaherpesviruses. mBio 6:e02213-14. doi:10.1128/mBio.02213-14. 25827418 PMC4453532

[24] Wilkinson GWG, Davison AJ, Tomasec P, Fielding CA, Aicheler R, Murrell I, Seirafian S, Wang ECY, Weekes M, Lehner PJ, Wilkie GS, Stanton RJ 2015 Human cytomegalovirus: taking the strain. Med Microbiol Immunol 204:273–284. doi:10.1007/s00430-015-0411-4. 25894764 PMC4439430

[25] Bowen CD, Renner DW, Shreve JT, Tafuri Y, Payne KM, Dix RD, Kinchington PR, Gatherer D, Szpara ML 2016 Viral forensic genomics reveals the relatedness of classic herpes simplex virus strains KOS, KOS63, and KOS79. Virology 492:179–186. doi:10.1016/j.virol.2016.02.013. 26950505 PMC5056906

[26] Greenbaum BD, Ghedin E 2015 Viral evolution: beyond drift and shift. Curr Opin Microbiol 26:109–115. doi:10.1016/j.mib.2015.06.015. 26189048

[27] Lee K, Kolb AW, Sverchkov Y, Cuellar JA, Craven M, Brandt CR 2015 Recombination analysis of herpes simplex virus 1 reveals a bias toward GC content and the inverted repeat regions. J Virol 89:7214–7223. doi:10.1128/JVI.00880-15. 25926637 PMC4473588

[28] Zell R, Taudien S, Pfaff F, Wutzler P, Platzer M, Sauerbrei A 2012 Sequencing of 21 varicella-zoster virus genomes reveals two novel genotypes and evidence of recombination. J Virol 86:1608–1622. doi:10.1128/JVI.06233-11. 22130537 PMC3264370

[29] Depledge DP, Gray ER, Kundu S, Cooray S, Poulsen A, Aaby P, Breuer J 2014 Evolution of cocirculating varicella-zoster virus genotypes during a chickenpox outbreak in Guinea-Bissau. J Virol 88:13936–13946. doi:10.1128/JVI.02337-14. 25275123 PMC4249134

[30] Norberg P, Depledge DP, Kundu S, Atkinson C, Brown J, Haque T, Hussaini Y, MacMahon E, Molyneaux P, Papaevangelou V, Sengupta N, Koay ESC, Tang JW, Underhill GS, Grahn A, Studahl M, Breuer J, Bergström T 2015 Recombination of globally circulating varicella-zoster virus. J Virol 89:7133–7146. doi:10.1128/JVI.00437-15. 25926648 PMC4473579

[31] Jeon JS, Won YH, Kim IK, Ahn JH, Shin OS, Kim JH, Lee CH 2016 Analysis of single nucleotide polymorphism among varicella-zoster virus and identification of vaccine-specific sites. Virology 496:277–286. doi:10.1016/j.virol.2016.06.017. 27376245

[32] Jensen NJ, Rivailler P, Tseng HF, Quinlivan ML, Radford K, Folster J, Harpaz R, LaRussa P, Jacobsen S, Scott Schmid D 2017 Revisiting the genotyping scheme for varicella-zoster viruses based on whole-genome comparisons. J Gen Virol 98:1434–1438. doi:10.1099/jgv.0.000772. 28613146 PMC5721928

[33] Sijmons S, Thys K, Mbong Ngwese M, Van Damme E, Dvorak J, Van Loock M, Li G, Tachezy R, Busson L, Aerssens J, Van Ranst M, Maes P 2015 High-throughput analysis of human cytomegalovirus genome diversity highlights the widespread occurrence of gene-disrupting mutations and pervasive recombination. J Virol 89:7673–7695. doi:10.1128/JVI.00578-15. PMC4505652 25972543

[34] Lassalle F, Depledge DP, Reeves MB, Brown AC, Christiansen MT, Tutill HJ, Williams RJ, Einer-Jensen K, Holdstock J, Atkinson C, Brown JR, van Loenen FB, Clark DA, Griffiths PD, Verjans GMGM, Schutten M, Milne RSB, Balloux F, Breuer J 2016 Islands of linkage in an ocean of pervasive recombination reveals two-speed evolution of human cytomegalovirus genomes. Virus Evol 2:vew017. doi:10.1093/ve/vew017. PMC6167919 30288299

[35] Norberg P, Tyler S, Severini A, Whitley R, Liljeqvist JÅ, Bergström T 25 7 2011 A genome-wide comparative evolutionary analysis of herpes simplex virus type 1 and varicella zoster virus. PLoS One doi:10.1371/journal.pone.0022527. PMC3143153 21799886

[36] Szpara ML, Gatherer D, Ochoa A, Greenbaum B, Dolan A, Bowden RJ, Enquist LW, Legendre M, Davison AJ 2014 Evolution and diversity in human herpes simplex virus genomes. J Virol 88:1209–1227. doi:10.1128/JVI.01987-13. 24227835 PMC3911644

[37] Koelle DM, Norberg P, Fitzgibbon MP, Russell RM, Greninger AL, Huang M-L, Stensland L, Jing L, Magaret AS, Diem K, Selke S, Xie H, Celum C, Lingappa JR, Jerome KR, Wald A, Johnston C 2017 Worldwide circulation of HSV-2 × HSV-1 recombinant strains. Sci Rep 7:44084. doi:10.1038/srep44084. 28287142 PMC5347006

[38] Burrel S, Boutolleau D, Ryu D, Agut H, Merkel K, Leendertz FH, Calvignac-Spencer S 2017 Ancient recombination events between human herpes simplex viruses. Mol Biol Evol 34:1713–1721. doi:10.1093/molbev/msx113. 28369565 PMC5455963

[39] Burrel S, Désiré N, Marlet J, Dacheux L, Seang S, Caumes E, Bourhy H, Agut H, Boutolleau D 2015 Genetic diversity within alphaherpesviruses: characterization of a novel variant of herpes simplex virus 2. J Virol 89:12273–12283. doi:10.1128/JVI.01959-15. 26401046 PMC4665239

[40] Wertheim JO, Smith MD, Smith DM, Scheffler K, Pond SLK 10 7 2014 Evolutionary origins of human herpes simplex viruses 1 and 2. Mol Biol Evol doi:10.1093/molbev/msu185. PMC4137711 24916030

[41] Loncoman CA, Vaz PK, Coppo MJ, Hartley CA, Morera FJ, Browning GF, Devlin JM 2017 Natural recombination in alphaherpesviruses: insights into viral evolution through full genome sequencing and sequence analysis. Infect Genet Evol 49:174–185. doi:10.1016/j.meegid.2016.12.022. 28017915

[42] Lee S-W, Markham PF, Coppo MJC, Legione AR, Markham JF, Noormohammadi AH, Browning GF, Ficorilli N, Hartley CA, Devlin JM 2012 Attenuated vaccines can recombine to form virulent field viruses. Science 337:188–188. doi:10.1126/science.1217134. 22798607

[43] Murphy E, Yu D, Grimwood J, Schmutz J, Dickson M, Jarvis MA, Hahn G, Nelson JA, Myers RM, Shenk TE 2003 Coding potential of laboratory and clinical strains of human cytomegalovirus. Proc Natl Acad Sci U S A 100:14976–14981. doi:10.1073/pnas.2136652100. 14657367 PMC299866

[44] Boyer M, Azza S, Barrassi L, Klose T, Campocasso A, Pagnier I, Fournous G, Borg A, Robert C, Zhang X, Desnues C, Henrissat B, Rossmann MG, La Scola B, Raoult D 2011 Mimivirus shows dramatic genome reduction after intraamoebal culture. Proc Natl Acad Sci U S A 108:10296–10301. doi:10.1073/pnas.1101118108. 21646533 PMC3121840

[45] Elde NC, Child SJ, Eickbush MT, Kitzman JO, Rogers KS, Shendure J, Geballe AP, Malik HS 2012 Poxviruses deploy genomic accordions to adapt rapidly against host antiviral defenses. Cell 150:831–841. doi:10.1016/j.cell.2012.05.049. 22901812 PMC3499626

[46] Alcami A 2003 Viral mimicry of cytokines, chemokines and their receptors. Nat Rev Immunol 3:36–50. doi:10.1038/nri980. 12511874

[47] McGeoch DJ, Davison AJ, Dolan A, Gatherer D, Sevilla-Reyes EE 2008 Molecular evolution of the Herpesvirales, p 447–475. *In* Origin and evolution of viruses Elsevier, Philadelphia, PA.

[48] Elde NC, Malik HS 2009 The evolutionary conundrum of pathogen mimicry. Nat Rev Microbiol 7:787–797. doi:10.1038/nrmicro2222. 19806153

[49] Rappoport N, Linial M 2012 Viral proteins acquired from a host converge to simplified domain architectures. PLoS Comput Biol 8:e1002364. doi:10.1371/journal.pcbi.1002364. 22319434 PMC3271019

[50] Aswad A, Katzourakis A 2015 Convergent capture of retroviral superantigens by mammalian herpesviruses. Nat Commun 6:8299. doi:10.1038/ncomms9299. 26400439 PMC4667437

[51] Morissette G, Flamand L 2010 Herpesviruses and chromosomal integration. J Virol 84:12100–12109. doi:10.1128/JVI.01169-10. 20844040 PMC2976420

[52] Xiao K, Yu Z, Li X, Li X, Tang K, Tu C, Qi P, Liao Q, Chen P, Zeng Z, Li G, Xiong W 2016 Genome-wide analysis of Epstein-Barr virus (EBV) integration and strain in C666-1 and Raji cells. J Cancer 7:214–224. doi:10.7150/jca.13150. 26819646 PMC4716855

[53] Kaufer BB, Jarosinski KW, Osterrieder N 2011 Herpesvirus telomeric repeats facilitate genomic integration into host telomeres and mobilization of viral DNA during reactivation. J Exp Med 208:605–615. doi:10.1084/jem.20101402. 21383055 PMC3058580

[54] Osterrieder N, Wallaschek N, Kaufer BB 2014 Herpesvirus genome integration into telomeric repeats of host cell chromosomes. Annu Rev Virol 1:215–235. doi:10.1146/annurev-virology-031413-085422. 26958721

[55] Wallaschek N, Sanyal A, Pirzer F, Gravel A, Mori Y, Flamand L, Kaufer BB 2016 The telomeric repeats of human herpesvirus 6A (HHV-6A) are required for efficient virus integration. PLoS Pathog 12:e1005666. doi:10.1371/journal.ppat.1005666. 27244446 PMC4887096

[56] Tweedy J, Spyrou M, Pearson M, Lassner D, Kuhl U, Gompels U 2016 Complete genome sequence of germline chromosomally integrated human herpesvirus 6A and analyses integration sites define a new human endogenous virus with potential to reactivate as an emerging infection. Viruses 8:E19. doi:10.3390/v8010019. 26784220 PMC4728579

[57] Gilbert C, Chateigner A, Ernenwein L, Barbe V, Bézier A, Herniou EA, Cordaux R 21 2 2014 Population genomics supports baculoviruses as vectors of horizontal transfer of insect transposons. Nat Commun doi:10.1038/ncomms4348. PMC3948050 24556639

[58] Gilbert C, Peccoud J, Chateigner A, Moumen B, Cordaux R, Herniou EA 2016 Continuous influx of genetic material from host to virus populations. PLoS Genet 12:e1005838. doi:10.1371/journal.pgen.1005838. 26829124 PMC4735498

[59] Griffiths A, Chen S, Horsburgh BC, Coen DM 2003 Translational compensation of a frameshift mutation affecting herpes simplex virus thymidine kinase is sufficient to permit reactivation from latency. J Virol 77:4703–4709. doi:10.1128/JVI.77.8.4703-4709.2003. 12663777 PMC152167

[60] Pan D, Coen DM 2012 Net −1 frameshifting on a noncanonical sequence in a herpes simplex virus drug-resistant mutant is stimulated by nonstop mRNA. Proc Natl Acad Sci U S A 109:14852–14857. doi:10.1073/pnas.1206582109. 22927407 PMC3443137

[61] Wang K, Mahalingam G, Hoover SE, Mont EK, Holland SM, Cohen JI, Straus SE 2007 Diverse herpes simplex virus type 1 thymidine kinase mutants in individual human neurons and ganglia. J Virol 81:6817–6826. doi:10.1128/JVI.00166-07. 17459924 PMC1933309

[62] Burrel S, Deback C, Agut H, Boutolleau D 2010 Genotypic characterization of UL23 thymidine kinase and UL30 DNA polymerase of clinical isolates of herpes simplex virus: natural polymorphism and mutations associated with resistance to antivirals. Antimicrob Agents Chemother 54:4833–4842. doi:10.1128/AAC.00669-10. 20733037 PMC2976144

[63] Lin Z, Wang X, Strong MJ, Concha M, Baddoo M, Xu G, Baribault C, Fewell C, Hulme W, Hedges D, Taylor CM, Flemington EK 2013 Whole-genome sequencing of the Akata and Mutu Epstein-Barr virus strains. J Virol 87:1172–1182. doi:10.1128/JVI.02517-12. 23152513 PMC3554088

[64] Arias C, Weisburd B, Stern-Ginossar N, Mercier A, Madrid AS, Bellare P, Holdorf M, Weissman JS, Ganem D 2014 KSHV 2.0: a comprehensive annotation of the Kaposi's sarcoma-associated herpesvirus genome using next-generation sequencing reveals novel genomic and functional features. PLoS Pathog 10:e1003847. doi:10.1371/journal.ppat.1003847. 24453964 PMC3894221

[65] Stern-Ginossar N, Weisburd B, Michalski A, Le VTK, Hein MY, Huang S-X, Ma M, Shen B, Qian S-B, Hengel H, Mann M, Ingolia NT, Weissman JS 2012 Decoding human cytomegalovirus. Science 338:1088–1093. doi:10.1126/science.1227919. 23180859 PMC3817102

[66] Tirosh O, Cohen Y, Shitrit A, Shani O, Le-Trilling VTK, Trilling M, Friedlander G, Tanenbaum M, Stern-Ginossar N 2015 The transcription and translation landscapes during human cytomegalovirus infection reveal novel host-pathogen interactions. PLoS Pathog 11:e1005288. doi:10.1371/journal.ppat.1005288. 26599541 PMC4658056

[67] O'Grady T, Wang X, Höner zu Bentrup K, Baddoo M, Concha M, Flemington EK 2016 Global transcript structure resolution of high gene density genomes through multi-platform data integration. Nucleic Acids Res 44:e145–e145. doi:10.1093/nar/gkw629. 27407110 PMC5062983

[68] Rutkowski AJ, Erhard F, L'Hernault A, Bonfert T, Schilhabel M, Crump C, Rosenstiel P, Efstathiou S, Zimmer R, Friedel CC, Dölken L 2015 Widespread disruption of host transcription termination in HSV-1 infection. Nat Commun 6:7126. doi:10.1038/ncomms8126. 25989971 PMC4441252

[69] Szpara ML, Parsons L, Enquist LW 2010 Sequence variability in clinical and laboratory isolates of herpes simplex virus 1 reveals new mutations. J Virol 84:5303–5313. doi:10.1128/JVI.00312-10. 20219902 PMC2863834

[70] Pfaff F, Groth M, Sauerbrei A, Zell R 24 8 2015 Genotyping of herpes simplex virus type 1 (HSV-1) by whole genome sequencing. J Gen Virol doi:10.1099/jgv.0.000589. 27558891

[71] Karamitros T, Harrison I, Piorkowska R, Katzourakis A, Magiorkinis G, Mbisa JL 2016 De novo assembly of human herpes virus type 1 (HHV-1) genome, mining of non-canonical structures and detection of novel drug-resistance mutations using short- and long-read next generation sequencing technologies. PLoS One 11:e0157600. doi:10.1371/journal.pone.0157600. 27309375 PMC4910999

[72] Petro CD, Weinrick B, Khajoueinejad N, Burn C, Sellers R, Jacobs WR, Herold BC 4 8 2016 HSV-2 ΔgD elicits FcγR-effector antibodies that protect against clinical isolates. JCI Insight doi:10.1172/jci.insight.88529. PMC4985247 27536733

[73] Newman RM, Lamers SL, Weiner B, Ray SC, Colgrove RC, Diaz F, Jing L, Wang K, Saif S, Young S, Henn M, Laeyendecker O, Tobian AAR, Cohen JI, Koelle DM, Quinn TC, Knipe DM 27 5 2015 Genome sequencing and analysis of geographically diverse clinical isolates of herpes simplex virus 2. J Virol doi:10.1128/JVI.01303-15. PMC4524243 26018166

[74] Kolb AW, Larsen IV, Cuellar JA, Brandt CR 2015 Genomic, phylogenetic, and recombinational characterization of herpes simplex virus 2 strains. J Virol 89:6427–6434. doi:10.1128/JVI.00416-15. 25855744 PMC4474301

[75] Johnston C, Magaret A, Roychoudhury P, Greninger AL, Cheng A, Diem K, Fitzgibbon MP, Huang M, Selke S, Lingappa JR, Celum C, Jerome KR, Wald A, Koelle DM 2017 Highly conserved intragenic HSV-2 sequences: results from next-generation sequencing of HSV-2 UL and US regions from genital swabs collected from 3 continents. Virology 510:90–98. doi:10.1016/j.virol.2017.06.031. 28711653 PMC5565707

[76] Minaya MA, Jensen T, Goll J, Korom M, Datla SH, Belshe RB, Morrison LA 20 9 2017 Molecular evolution of herpes simplex virus 2 complete genomes: comparison between primary and recurrent infections. J Virol doi:10.1128/JVI.00942-17. PMC5686715 28931680

[77] Hage E, Wilkie GS, Linnenweber-Held S, Dhingra A, Suárez NM, Schmidt JJ, Kay-Fedorov PC, Mischak-Weissinger E, Heim A, Schwarz A, Schulz TF, Davison AJ, Ganzenmueller T 2017 Characterization of human cytomegalovirus genome diversity in immunocompromised hosts by whole-genome sequencing directly from clinical specimens. J Infect Dis 215:1673–1683. doi:10.1093/infdis/jix157. 28368496

[78] Palser AL, Grayson NE, White RE, Corton C, Correia S, Ba Abdullah MM, Watson SJ, Cotten M, Arrand JR, Murray PG, Allday MJ, Rickinson AB, Young LS, Farrell PJ, Kellam P 2015 Genome diversity of Epstein-Barr virus from multiple tumor types and normal infection. J Virol 89:5222–5237. doi:10.1128/JVI.03614-14. 25787276 PMC4442510

[79] Tu C, Zeng Z, Qi P, Li X, Yu Z, Guo C, Xiong F, Xiang B, Zhou M, Gong Z, Liao Q, Yu J, He Y, Zhang W, Li X, Li Y, Li G, Xiong W 2017 Genome-wide analysis of 18 Epstein-Barr viruses isolated from primary nasopharyngeal carcinoma biopsy specimens. J Virol 91:e00301-17. doi:10.1128/JVI.00301-17. 28637758 PMC5553176

[80] Olp LN, Jeanniard A, Marimo C, West JT, Wood C 2015 Whole-genome sequencing of Kaposi's sarcoma-associated herpesvirus from Zambian Kaposi's sarcoma biopsy specimens reveals unique viral diversity. J Virol 89:12299–12308. doi:10.1128/JVI.01712-15. 26423952 PMC4665246

[81] Depledge DP, Palser AL, Watson SJ, Lai IY-C, Gray ER, Grant P, Kanda RK, Leproust E, Kellam P, Breuer J 2011 Specific capture and whole-genome sequencing of viruses from clinical samples. PLoS One 6:e27805. doi:10.1371/journal.pone.0027805. 22125625 PMC3220689

[82] Duggan AT, Perdomo MF, Piombino-Mascali D, Marciniak S, Poinar D, Emery MV, Buchmann JP, Duchêne S, Jankauskas R, Humphreys M, Golding GB, Southon J, Devault A, Rouillard J-M, Sahl JW, Dutour O, Hedman K, Sajantila A, Smith GL, Holmes EC, Poinar HN 2016 17th century variola virus reveals the recent history of smallpox. Curr Biol 26:3407–3412. doi:10.1016/j.cub.2016.10.061. 27939314 PMC5196022

[83] Posada-Cespedes S, Seifert D, Beerenwinkel N 28 9 2016 Recent advances in inferring viral diversity from high-throughput sequencing data. Virus Res doi:10.1016/j.virusres.2016.09.016. 27693290

[84] Oldfield LM, Grzesik P, Voorhies AA, Alperovich N, MacMath D, Najera CD, Chandra DS, Prasad S, Noskov VN, Montague MG, Friedman RM, Desai PJ, Vashee S 19 9 2017 Genome-wide engineering of an infectious clone of herpes simplex virus type 1 using synthetic genomics assembly methods. Proc Natl Acad Sci U S A doi:10.1073/pnas.1700534114. PMC5651731 28928148

[85] Tombácz D, Csabai Z, Szűcs A, Balázs Z, Moldován N, Sharon D, Snyder M, Boldogkői Z 20 6 2017 Long-read isoform sequencing reveals a hidden complexity of the transcriptional landscape of herpes simplex virus type 1. Front Microbiol doi:10.3389/fmicb.2017.01079. PMC5476775 28676792

[86] Renzette N, Kowalik TF, Jensen JD 2016 On the relative roles of background selection and genetic hitchhiking in shaping human cytomegalovirus genetic diversity. Mol Ecol 25:403–413. doi:10.1111/mec.13331. 26211679 PMC4706826

[87] Pokalyuk C, Renzette N, Irwin KK, Pfeifer SP, Gibson L, Britt WJ, Yamamoto AY, Mussi-Pinhata MM, Kowalik TF, Jensen JD 8 2 2017 Characterizing human cytomegalovirus reinfection in congenitally infected infants: an evolutionary perspective. Mol Ecol doi:10.1111/mec.13953. 27988973

[88] Köser CU, Ellington MJ, Cartwright EJP, Gillespie SH, Brown NM, Farrington M, Holden MTG, Dougan G, Bentley SD, Parkhill J, Peacock SJ 2012 Routine use of microbial whole genome sequencing in diagnostic and public health microbiology. PLoS Pathog 8:e1002824. doi:10.1371/journal.ppat.1002824. 22876174 PMC3410874

[89] Houldcroft CJ, Beale MA, Breuer J 2017 Clinical and biological insights from viral genome sequencing. Nat Rev Microbiol 15:183–192. doi:10.1038/nrmicro.2016.182. 28090077 PMC7097211

[90] Holland TC, Marlin SD, Levine M, Glorioso J 1983 Antigenic variants of herpes simplex virus selected with glycoprotein-specific monoclonal antibodies. J Virol 45:672–682. 6187935 10.1128/jvi.45.2.672-682.1983 PMC256462

[91] Dix RD, McKendall RR, Baringer JR 1983 Comparative neurovirulence of herpes simplex virus type 1 strains after peripheral or intracerebral inoculation of BALB/c mice. Infect Immun 40:103–112. 6299955 10.1128/iai.40.1.103-112.1983 PMC264823

[92] Norberg P, Bergstrom T, Rekabdar E, Lindh M 2004 Phylogenetic analysis of clinical herpes simplex virus type 1 isolates identified three genetic groups and recombinant viruses. J Virol 78:10755–10764. doi:10.1128/JVI.78.19.10755-10764.2004. 15367642 PMC516408

[93] Sato A, Linehan MM, Iwasaki A 2006 Dual recognition of herpes simplex viruses by TLR2 and TLR9 in dendritic cells. Proc Natl Acad Sci U S A 103:17343–17348. doi:10.1073/pnas.0605102103. 17085599 PMC1859932

[94] Colgrove RC, Liu X, Griffiths A, Raja P, Deluca NA, Newman RM, Coen DM, Knipe DM 2016 History and genomic sequence analysis of the herpes simplex virus 1 KOS and KOS1.1 sub-strains. Virology 487:215–221. doi:10.1016/j.virol.2015.09.026. 26547038 PMC4679709

[95] Pandey U, Bell AS, Renner DW, Kennedy DA, Shreve JT, Cairns CL, Jones MJ, Dunn PA, Read AF, Szpara ML 2016 DNA from dust: comparative genomics of large DNA viruses in field surveillance samples. mSphere 1:e00132-16. doi:10.1128/mSphere.00132-16. 27747299 PMC5064450

[96] Breuer J, Grose C, Norberg P, Tipples G, Schmid DS 2010 A proposal for a common nomenclature for viral clades that form the species varicella-zoster virus: summary of VZV Nomenclature Meeting 2008, Barts and the London School of Medicine and Dentistry, 24–25 July 2008. J Gen Virol 91:821–828. doi:10.1099/vir.0.017814-0. 20071486 PMC2888159

[97] Kuhn JH, Bao Y, Bavari S, Becker S, Bradfute S, Brister JR, Bukreyev AA, Chandran K, Davey RA, Dolnik O, Dye JM, Enterlein S, Hensley LE, Honko AN, Jahrling PB, Johnson KM, Kobinger G, Leroy EM, Lever MS, Mühlberger E, Netesov SV, Olinger GG, Palacios G, Patterson JL, Paweska JT, Pitt L, Radoshitzky SR, Saphire EO, Smither SJ, Swanepoel R, Towner JS, van der Groen G, Volchkov VE, Wahl-Jensen V, Warren TK, Weidmann M, Nichol ST 2013 Virus nomenclature below the species level: a standardized nomenclature for natural variants of viruses assigned to the family Filoviridae. Arch Virol 158:301–311. doi:10.1007/s00705-012-1454-0. 23001720 PMC3535543

[98] Wan Y, Renner DW, Albert I, Szpara ML 28 4 2015 VirAmp: a galaxy-based viral genome assembly pipeline. GigaScience doi:10.1186/s13742-015-0060-y. PMC4410580 25918639

[99] Giardine B, Riemer C, Hardison RC, Burhans R, Elnitski L, Shah P, Zhang Y, Blankenberg D, Albert I, Taylor J, Miller W, Kent WJ, Nekrutenko A 2005 Galaxy: a platform for interactive large-scale genome analysis. Genome Res 15:1451–1455. doi:10.1101/gr.4086505. 16169926 PMC1240089

[100] Blankenberg D, Von Kuster G, Coraor N, Ananda G, Lazarus R, Mangan M, Nekrutenko A, Taylor J 2010 Galaxy: a web-based genome analysis tool for experimentalists. Curr Protoc Mol Biol Chapter 19:Unit 19.10.1–21. doi:10.1002/0471142727.mb1910s89. PMC4264107 20069535

[101] Kronenberg ZN, Osborne EJ, Cone KR, Kennedy BJ, Domyan ET, Shapiro MD, Elde NC, Yandell M 2015 Wham: identifying structural variants of biological consequence. PLoS Comput Biol 11:e1004572. doi:10.1371/journal.pcbi.1004572. 26625158 PMC4666669

[102] Jordan TC, Burnett SH, Carson S, Caruso SM, Clase K, DeJong RJ, Dennehy JJ, Denver DR, Dunbar D, Elgin SCR, Findley AM, Gissendanner CR, Golebiewska UP, Guild N, Hartzog GA, Grillo WH, Hollowell GP, Hughes LE, Johnson A, King RA, Lewis LO, Li W, Rosenzweig F, Rubin MR, Saha MS, Sandoz J, Shaffer CD, Taylor B, Temple L, Vazquez E, Ware VC, Barker LP, Bradley KW, Jacobs-Sera D, Pope WH, Russell DA, Cresawn SG, Lopatto D, Bailey CP, Hatfull GF 2014 A broadly implementable research course in phage discovery and genomics for first-year undergraduate students. mBio 5:e01051-13. doi:10.1128/mBio.01051-13. 24496795 PMC3950523

[103] Hatfull GF, Racaniello V 2014 PHIRE and *TWiV*: experiences in bringing virology to new audiences. Annu Rev Virol 1:37–53. doi:10.1146/annurev-virology-031413-085449. 26958714

[104] Fraser CM, Eisen JA, Nelson KE, Paulsen IT, Salzberg SL 2002 The value of complete microbial genome sequencing (you get what you pay for). J Bacteriol 184:6403–6405. doi:10.1128/JB.184.23.6403-6405.2002. 12426324 PMC135419

[105] Reuter JA, Spacek DV, Snyder MP 2015 High-throughput sequencing technologies. Mol Cell 58:586–597. doi:10.1016/j.molcel.2015.05.004. 26000844 PMC4494749

[106] Danaher RJ, Fouts DE, Chan AP, Choi Y, DePew J, McCorrison JM, Nelson KE, Wang C, Miller CS 13 10 2016 HSV-1 clinical isolates with unique in vivo and in vitro phenotypes and insight into genomic differences. J Neurovirol doi:10.1007/s13365-016-0485-9. 27739035

[107] Kolb AW, Adams M, Cabot EL, Craven M, Brandt CR 2011 Multiplex sequencing of seven ocular herpes simplex virus type-1 genomes: phylogeny, sequence variability, and SNP distribution. Invest Ophthalmol Vis Sci 52:9061–9073. doi:10.1167/iovs.11-7812. 22016062 PMC3231845

[108] Suenaga T, Kohyama M, Hirayasu K, Arase H 2014 Engineering large viral DNA genomes using the CRISPR-Cas9 system: editing of viral genomes with CRISPR-Cas9. Microbiol Immunol 58:513–522. doi:10.1111/1348-0421.12180. 25040500 PMC7168497

[109] Russell TA, Stefanovic T, Tscharke DC 2015 Engineering herpes simplex viruses by infection-transfection methods including recombination site targeting by CRISPR/Cas9 nucleases. J Virol Methods 213:18–25. doi:10.1016/j.jviromet.2014.11.009. 25479355

[110] van Diemen FR, Kruse EM, Hooykaas MJG, Bruggeling CE, Schürch AC, van Ham PM, Imhof SM, Nijhuis M, Wiertz EJHJ, Lebbink RJ 2016 CRISPR/Cas9-mediated genome editing of herpesviruses limits productive and latent infections. PLoS Pathog 12:e1005701. doi:10.1371/journal.ppat.1005701. 27362483 PMC4928872

[111] Dutilh BE, Backus L, Edwards RA, Wels M, Bayjanov JR, van Hijum SAFT 2013 Explaining microbial phenotypes on a genomic scale: GWAS for microbes. Brief Funct Genomics 12:366–380. doi:10.1093/bfgp/elt008. 23625995 PMC3743258

[112] Power RA, Parkhill J, de Oliveira T 2017 Microbial genome-wide association studies: lessons from human GWAS. Nat Rev Genet 18:41–50. doi:10.1038/nrg.2016.132. 27840430

[113] Kolb AW, Lee K, Larsen I, Craven M, Brandt CR 2016 Quantitative trait locus based virulence determinant mapping of the HSV-1 genome in murine ocular infection: genes involved in viral regulatory and innate immune networks contribute to virulence. PLoS Pathog 12:e1005499. doi:10.1371/journal.ppat.1005499. 26962864 PMC4786273

[114] Lee K, Kolb AW, Larsen I, Craven M, Brandt CR 2016 Mapping murine corneal neovascularization and weight loss virulence determinants in the herpes simplex virus 1 genome and the detection of an epistatic interaction between the UL and IRS/US regions. J Virol 90:8115–8131. doi:10.1128/JVI.00821-16. 27384650 PMC5008079

[115] Roizman B, Campadelli-Fiume G 2007 Alphaherpes viral genes and their functions, p 70–83. *In* Human Herpesviruses: Biology, Therapy, and Immunoprophylaxis . Cambridge University Press, Cambridge, United Kingdom. 21348071

[116] Dunn W, Chou C, Li H, Hai R, Patterson D, Stolc V, Zhu H, Liu F 2003 Functional profiling of a human cytomegalovirus genome. Proc Natl Acad Sci U S A 100:14223–14228. doi:10.1073/pnas.2334032100. 14623981 PMC283573

[117] Arav-Boger R 2015 Strain variation and disease severity in congenital cytomegalovirus infection. Infect Dis Clin North Am 29:401–414. doi:10.1016/j.idc.2015.05.009. 26154664 PMC4552582

[118] Mechelli R, Manzari C, Policano C, Annese A, Picardi E, Umeton R, Fornasiero A, D'erchia AM, Buscarinu MC, Agliardi C, Annibali V, Serafini B, Rosicarelli B, Romano S, Angelini DF, Ricigliano VA, Buttari F, Battistini L, Centonze D, Guerini FR, D'Alfonso S, Pesole G, Salvetti M, Ristori G 2015 Epstein-Barr virus genetic variants are associated with multiple sclerosis. Neurology 84:1362–1368. doi:10.1212/WNL.0000000000001420. 25740864 PMC4388746

[119] Zhang E, Bell AJ, Wilkie GS, Suárez NM, Batini C, Veal CD, Armendáriz-Castillo I, Neumann R, Cotton VE, Huang Y, Porteous DJ, Jarrett RF, Davison AJ, Royle NJ 2017 Inherited chromosomally integrated human herpesvirus 6 genomes are ancient, intact, and potentially able to reactivate from telomeres. J Virol 91:e01137-17. doi:10.1128/JVI.01137-17. 28835501 PMC5660504

